# Roles of circRNAs in regulating the tumor microenvironment

**DOI:** 10.1007/s12032-023-02194-4

**Published:** 2023-10-11

**Authors:** Tao Liu, Kaijun Long, Zhengfeng Zhu, Yongxiang Song, Cheng Chen, Gang Xu, Xixian Ke

**Affiliations:** Department of Thoracic Surgery, Affiliated Hospital of Zunyi Medical University, 149 Dalian Road, Zunyi, 563000 Guizhou China

**Keywords:** CircRNAs, TME, ECM, Immune

## Abstract

CircRNAs, a type of non-coding RNA widely present in eukaryotic cells, have emerged as a prominent focus in tumor research. However, the functions of most circRNAs remain largely unexplored. Known circRNAs exert their regulatory roles through various mechanisms, including acting as microRNA sponges, binding to RNA-binding proteins, and functioning as transcription factors to modulate protein translation and coding. Tumor growth is not solely driven by gene mutations but also influenced by diverse constituent cells and growth factors within the tumor microenvironment (TME). As crucial regulators within the TME, circRNAs are involved in governing tumor growth and metastasis. This review highlights the role of circRNAs in regulating angiogenesis, matrix remodeling, and immunosuppression within the TME. Additionally, we discuss current research on hypoxia-induced circRNAs production and commensal microorganisms’ impact on the TME to elucidate how circRNAs influence tumor growth while emphasizing the significance of modulating the TME.

## Background

Cancer is often referred to as “a wound that never heals”, characterized by the uncontrolled proliferation of malignant cells alongside impaired immune system function. Abnormal expression or mutation of oncogenes/cancer suppressor genes leads to heterogeneity among normal cells, transforming them into malignancy [1]. In the early stages of tumor development, lymphocytes recognize surface antigens expressed by cancer cells and eliminate them through immune responses. To evade immune surveillance effectively, cancer cells shed surface antigens while secreting cytokines that recruit immune-suppressor cells for suppressing immune responses [2, 3].

The “seed and soil” hypothesis, proposed by Stephen Paget in 1889, suggests that the interaction between tumor cells (the seed) and their microenvironment (the soil) is crucial [4]. The TME is composed of various cellular components such as immune cells, tumor-associated endothelial cells (CAEs), tumor-associated fibroblasts (CAFs), pericytes, etc., as well as non-cellular components including cytokines, growth factors, metabolic substances, and extracellular matrix (ECM) proteins [5]. Rapid tumor growth triggers environmental changes, such as hypoxia and acidosis, which disrupt coordinated cellular interactions, leading to ECM remodeling, the induction of angiogenesis, and the inhibition of immune response. Consequently, a heterogeneous ecological environment conducive to cancer development is established [5, 6].

**Fig. 1:**
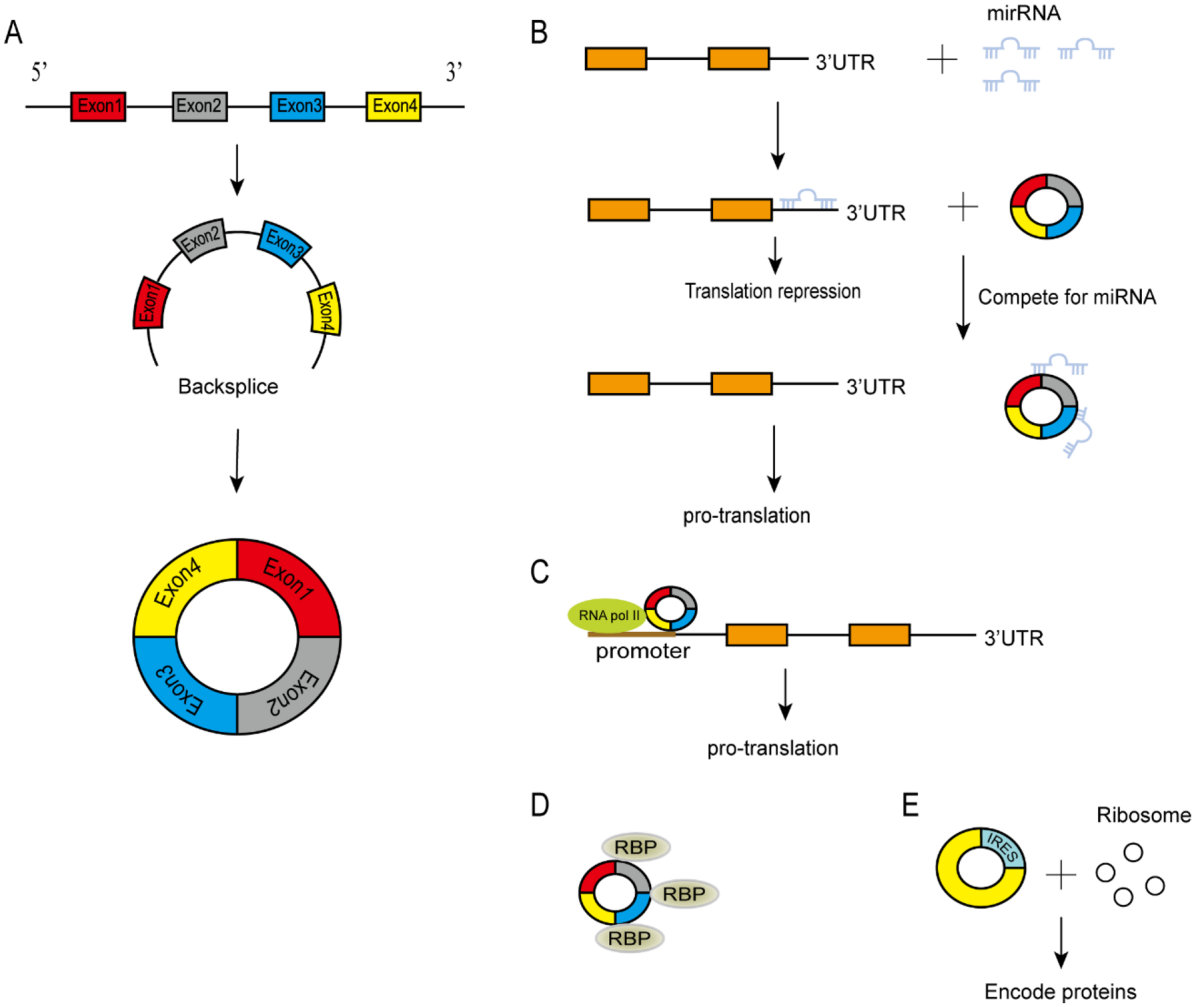
CircRNAs’ biogenesis and functions: **A** CircRNAs are formed by backsplicing linear RNA molecules at their 5′ and 3′ ends. **B** CircRNAs function as competitive endogenous RNA molecules, sequestering miRNAs. **C **CircRNAs regulate gene transcription and expression. **D** Interact with RNA-binding proteins. **E** CircRNAs can encode proteins driven by IRES

In recent years, circRNAs have become a research hotspot in cancer studies. There are complex regulatory interactions between circRNAs and TME components [7–10]. These findings provide ideas and theoretical basis for developing new cancer treatment methods. This review highlights the role of circRNAs in regulating angiogenesis, matrix remodeling, and immunosuppression within the TME. Additionally, we discuss current research on hypoxia-induced circRNAs production and commensal microorganisms’ impact on the TME to elucidate how circRNAs influence tumor growth while emphasizing the significance of modulating the TME (Fig. 1).

### CircRNAs functions

**Fig. 2:**
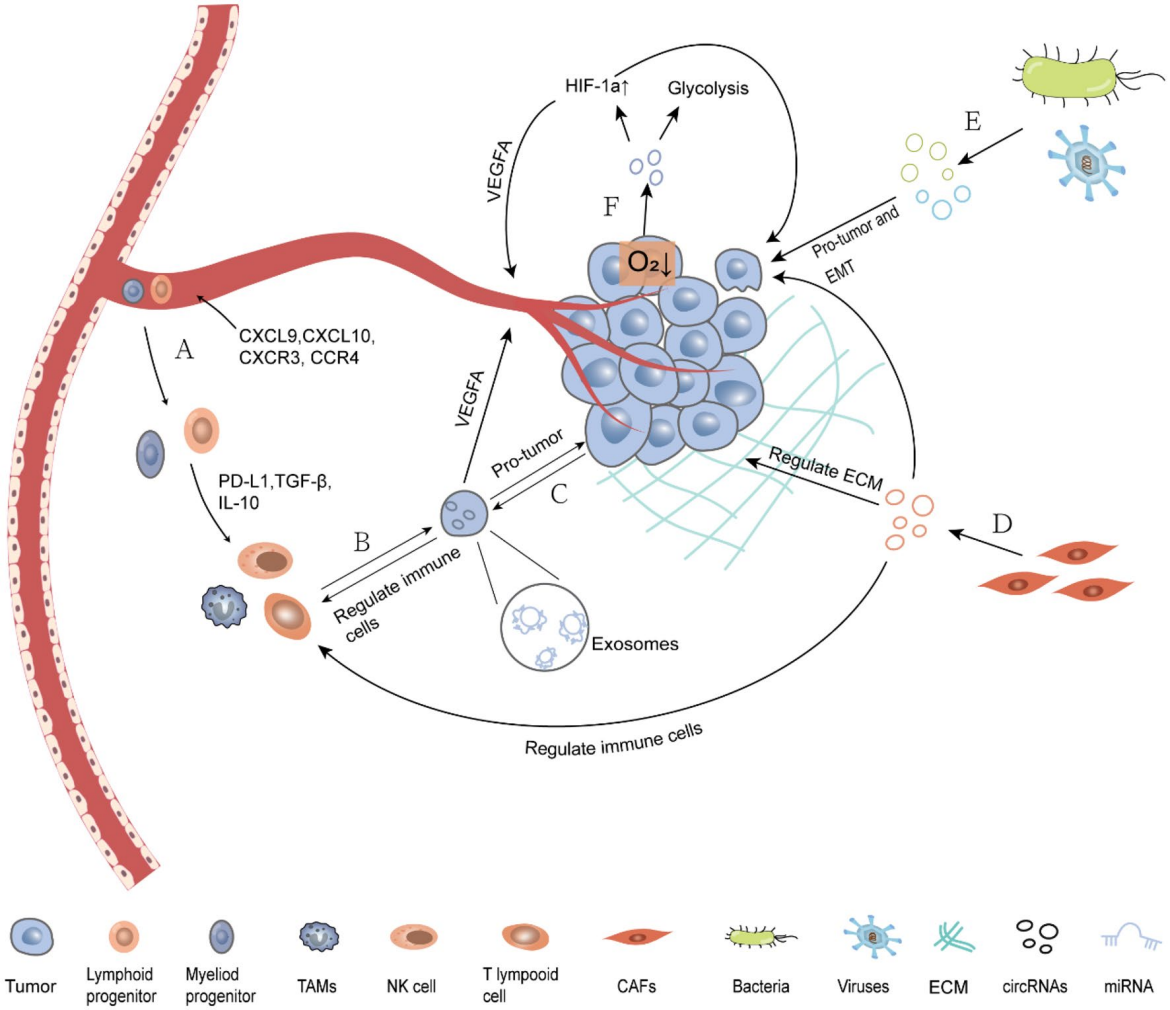
Roles of circRNAs in regulating the tumor microenvironment. **A** The chemotactic factors in the TME recruit lymphocytes and myeloid cells, which then differentiate into immunosuppressive cells under the influence of cytokines. **B** The exosomal circRNAs derived from immune-suppressive cells promote tumor growth and regulate angiogenesis. **C** CircRNAs derived from tumors induce polarization of macrophages and suppress the tumor-killing activity of TILs. **D** The exosomal circRNAs derived from CAFs promote tumor growth, regulate extracellular matrix remodeling, and modulate immune cell activity. **E** CircRNAs derived from tumor symbiotic microorganisms promote tumor growth and EMT. **F** Under hypoxic conditions, tumor-derived circRNAs regulate glucose metabolism through the glycolytic pathway, promote tumor growth and angiogenesis by modulating the expression of HIF-1a

Compared to traditional linear RNAs, circRNAs are characterized by their closed-loop structure, which confers resistance to RNA exonucleases and enables abundant and stable expression in cells and body fluids. With the development of RNA sequencing, thousands of circRNAs have been found in mammalian cells. Initially thought to be by-products of shearing, circRNAs play an important role in regulating the onset and development of many diseases [11, 12]. Current studies show that their functions can be divided into four parts: (1) Acting as miRNA sponges or repositories [13, 14]. (2) Interacting with RNA-binding proteins [15, 16]. (3) Functioning as translated proteins/peptides [17, 18]. And (4) regulating gene transcription and expression [19, 20]. A majority of dysregulated circRNAs have emerged as crucial regulators in cancer progression by modulating numerous cancer-associated molecules, thereby promoting tumorigenesis, suppressing tumor immunity, inducing angiogenesis, facilitating invasion and metastasis. Furthermore, circRNAs exhibit aberrant expression patterns in various diseases and govern disease progression encompassing cardiovascular diseases, autoimmune disorders, and inflammation [21–23] (Fig. 2).

### CircRNAs regulate angiogenesis in the tumor microenvironment

Angiogenesis, a hallmark of cancer, facilitates the provision of adequate nutrition and removal of metabolic wastes from tumors. Stimulated by hypoxia and nutrient deficiency, tumor cells release vascular endothelial growth factor (VEGF) to promote germination and proliferation of endothelial cells [24]. Additionally, constituent cells of the TME, including TAMs, mesenchymal stem cells (MSCs), and CAFs, contribute to tumor angiogenesis by releasing substantial amounts of pro-angiogenic signals [25, 26].

The main mechanism by which circRNAs regulate angiogenesis is through the ceRNA network, with VEGF being one of its major targets. For instance, in colorectal cancer, circ_0056618 enhances angiogenesis by binding to miR-206 and subsequently upregulating CXCR4 and VEGFA expression [27]. In glioblastoma stem cells (GSCs), circARF1 upregulates ISL2 expression via sponge adsorption of miR-342-3p, which in turn regulates VEGFA expression. This promotes endothelial cell proliferation and angiogenesis through the VEGFA-mediated ERK signaling pathway [28]. Moreover, up-regulation of circSHKBP1 exosomes in gastric cancer enhances VEGF mRNA stability by regulating human antigen R (HUR) expression. Consequently, this promotes gastric cancer angiogenesis [29].

Furthermore, circRNAs also regulate angiogenesis through the modulation of other gene expressions. In colorectal cancer (CRC), circ3823 acts as a competitive endogenous RNA for miR-30c-5p to alleviate its inhibitory effect on TCF7. As a result, MYC and CCND1 are upregulated leading to CRC progression and angiogenesis [30].Circ-CCAC1 derived from extracellular vesicles disrupts endothelial barrier integrity when translocated onto endothelial monolayers thereby inducing angiogenesis in cholangiocarcinoma (CCA) [31]. In osteosarcoma, the YY1 transcription factor induces upregulation of circFIRRE and partially increases mRNA and protein levels of LUZP1 by adsorbing miR-486-3p and miR-1225-5p, promoting tumor cell growth and angiogenesis, thereby driving primary osteosarcoma progression and lung metastasis [32].

Notably, cancer cells can obtain blood supply through mechanisms independent of endothelial cell proliferation such as vascular co-option (VC) and vasculogenic mimicry (VM) [33]. These two processes provide an alternative blood supply for neovascularization in cancer. Therefore, they are considered the main reason for anti-angiogenic therapy failure in some cancers [34, 35]. CircRNAs may also have regulatory roles in VC and VM generation processes [36]. For instance, exosomal circRNA-100,338 affects the cell proliferation and VM-forming ability of human umbilical vein endothelial cells (HUVEC) to promote hepatocellular carcinoma metastasis both in vitro and in vivo experiments [37]. Additionally, circRNA-000284 was significantly upregulated in cervical cancer cells regulating SNAIL2 expression by directly targeting miR-506 the transcription factor SNAIL2 positively regulates L1CAM to promote cancer cell attachment to preexisting blood vessels spreading along them [38]. Moreover, cancer cells utilize the flexor foot, an actin-based cytoplasmic extension with high expression of CDC42 and CD44, to migrate along the outer surface of blood vessels. Silencing this mechanism inhibits vascular co-option (VC) by disrupting contact with pericytes. In renal clear cell carcinoma, androgen receptor promotes VC through modulation of circHIAT1/miR-195-5p/29a-3p/29c-3p/CDC42 signaling [39] (Table 1).

**Table 1:** Summary of circRNAs and their functions in regulating angiogenesis

circRNAs	Functions	Expression	Targets	Origin	PMID
circ_0056618	Angiogenesis	Upregulate	miR-206/ CXCR4/VEGF-A	Colorectal cancer	32373955
circARF1	Angiogenesis	Upregulate	miR-342-3p/ISL2	Glioblastoma	32894165
circSHKBP1	Angiogenesis	Upregulate	miR-582-3p/ HUR/VEGF	Gastric cancer	32600329
circ-CCAC1	Angiogenesis	Upregulate	GRB2	Cholangiocarci-noma	32750152
circ3823	Angiogenesis	Upregulate	miR-30c-5p/ TCF7	Colorectal cancer	34172072
circFIRRE	Angiogenesis	Upregulate	miR-486-3p/ LUZP1	Osteosarcoma	35986280
circ-100,338	Angiogenesis	Upregulate	/	Hepatocellular carcinoma	31973767
circRNA-000284	Angiogenesis	Upregulate	miR-506/SNAIL2	Cervical cancer	29511454
circHIAT1	Angiogenesis	Upregulate	miR-195-5p/ 29a-3p/29c-3p/ CDC42	Clear cell renal clear cell carcinoma	28089832
circ_TNFRSF21	Angiogenesis	Upregulate	miR-3619-5p/ ROCK	Cutaneous squamous cell carcinoma	35593591

### CircRNAs and extracellular matrix remodeling

The extracellular matrix (ECM), composed of various fibrous components (e.g., collagen, fibronectin, and elastin) and non-fibrous molecules (e.g., proteoglycans, hyaluronic acid, and glycoproteins), plays a crucial role as a tissue barrier against tumor invasion and metastasis. In the context of tumors, the precise regulation of ECM homeostasis is disrupted by cancer cells, cancer-associated fibroblasts (CAFs), immune cells, and other stromal cells. Perturbation and dysregulation of the ECM elicit diverse physiological signals that facilitate cancer cell proliferation and invasion [40, 41].

CircRNAs have been identified to modulate the expression of ECM components such as collagen proteins (e.g., COL5A1 and COL1A1) in multiple types of cancers. For instance, in breast cancer,circACAP2 targets specific miRNAs to promote breast tumor progression through regulating the expression of adsorbed miRNA-targeted genes like collagen proteins COL5A [42]. Similarly, in nasopharyngeal carcinoma, circ_0000523 exerts its function by sequestering miRNAs to regulate the expression levels of target genes including collagen proteins like COL1A [43]. In oral squamous cell carcinoma, hsa_circRNA_0001971 facilitates disease progression by adsorbing miR-186-5p to modulate the expression of fibronectin FNDC3B [44]. Additionally, the circ-FNDC3B derived from the FNDC3B gene plays a crucial role in various tumors such as gastric cancer, esophageal cancer, and colorectal cancer [45–47], it promotes tumor epithelial-mesenchymal transition (EMT), invasion, and metastasis by regulating E-cadherin and CD44. Furthermore, hsa_circ_0000285 and circ-CAMK2A enhance tumor growth and metastasis via upregulating FN1 expression through targeting specific adsorbed miRNAs in Gastric cancer and lung cancer respectively [48, 49]. Syndecan (SDC), an integral membrane protein involved in intercellular adhesion, signal transduction, and cell-matrix interactions via its extracellular matrix protein receptor, exhibits abnormal expression patterns across various cancers [50–52]. Circ_RPPH1 enhances SDC1 expression to drive glioma malignancy via sequestration of miR-627‐5p/miR‐663a [53], whereas circ_0058063 facilitates thyroid cancer progression by sponging miR‐330‐3p/SDC4 axis [54]. Additionally, hyaluronic acid(HA), which serves as a critical constituent within ECM regulating tissue stiffness, maintaining homeostasis, and functioning as a signaling molecule for multiple cellular subtypes, is known to promote tumorigenesis including proliferation, migration, invasion, and drug resistance via ECM remodeling within diverse neoplastic contexts [55–57]. In papillary thyroid cancer,circ_102002 acts as a miR-488-3p sponge, leading to the upregulation of hyaluronic acid synthetase 2 (HAS2), which in turn promotes EMT and cell migration [58].

Tumor cells and cancer-associated fibroblasts (CAFs) co-secrete various matrix-remodeling enzymes, including MMPs, ADAMs, and ADAMTs, which degrade extracellular matrix (ECM) components and release stromal factors and growth factors to promote cancer cell growth, metastasis, and angiogenesis [41].CircRNAs play crucial roles in regulating the expression of MMPs. For instance, circMMP1 promotes colorectal cancer growth and metastasis by sponging miR-1238 to upregulate the expression of MMP1/MMP2/MMP9 [59]. Hsa_circRNA_101996 enhances gastric cancer progression by upregulating MMP-2/MMP-9 via the miR-143/TET2 pathway [60]. High expression of ciRS-7 maintains the migratory and invasive properties of triple-negative breast cancer cells by acting as a competing endogenous RNA for miR-1299 to increase the expression of MMPs [61].

### CircRNAs and CAFs

Cancer-associated fibroblasts (CAFs) play a pivotal role in the tumor microenvironment, engaging in extensive interactions with cancer cells and exerting influence on other TME components, including the extracellular matrix, angiogenesis, and immune infiltration. Fibroblasts are activated to transform into CAFs by various stimuli derived from tumors and immune-infiltrating cells, such as transforming growth factor β (TGF-β) family ligands, lysophosphatidic acid (LPA), fibroblast growth factor (FGF), platelet-derived growth factor (PDGF), interleukin-1 (IL-1), IL-6, and granulin [62, 63]. The abundance of CAFs is closely associated with the prognosis of different human tumors, while also governing therapeutic efficacy and serving as potential therapeutic targets themselves [64]. CircCUL2 is specifically expressed in CAFs. Upregulation of circCUL2 expression induces an activated CAF phenotype, which functions as a ceRNA and modulates the miR-203a-3p/MyD88/NF-κB/IL6 axis, then promotes the progression of pancreatic ductal adenocarcinoma (PDAC) by secreting IL-6 [65]. Cytokines derived from CAFs play a crucial role in tumor progression and metastasis by modulating the expression of circRNAs. In hepatocellular carcinoma, upregulated expression of circUBAP2 is observed in tumor tissues stimulated by CXCL11 secreted from CAFs. CircUBAP2 enhances the levels of IFIT1/3 and facilitates the expression of IL-17 and IL-1β through miR-4756 targeting, thereby enhancing the migratory capacity of hepatocellular carcinoma cells [66].

Additionally, CAFs play a crucial role in promoting tumor drug resistance, and their mechanism of resistance may be linked to the aberrant expression of circRNAs. Specifically, circZFR is highly expressed in CAFs and exosomes derived from CAFs. Elevated levels of circZFR have been shown to inhibit the STAT3/NF-κB pathway, thereby enhancing cisplatin resistance and promoting tumor growth [67]. In another study, it was found that CAFs can induce cancer stemness and gemcitabine resistance through leukemia inhibitory factor (LIF), which is promoted by circFARP1 specifically expressed in CAFs via direct binding to caveolin 1 (CAV1). Moreover, high levels of circFARP1 were positively associated with poorer survival and gemcitabine chemoresistance in pancreatic ductal adenocarcinoma patients due to its upregulation of LIF through sponge adsorption of miR-660-3p [68] (Table 2).

**Table 2:** Summary of circRNAs and their functions in regulating ECM

circRNAs	Functions	Expression	Targets	Origin	PMID
circACAP2	Matrix remodeling	Upregulate	miR-29a/b-3p/ COL5A1	Breast cancer	31,863,774
circ_0000523	Matrix remodeling	Upregulate	miR-1184/ COL1A1/PI3K/ Akt	Nasopharyngeal carcinoma	35,759,163
hsa_circRNA_ 0001971	Matrix remodeling	Upregulate	miR-186-5p/ FNDC3B	Oral squamous cell carcinoma	35,060,189
circ-FNDC3B	Matrix remodeling	Upregulate		esophageal	31,949,813
hsa_circ_0000285	Matrix remodeling	Upregulate	miR-1278/FN1	Gastric cancer	35,535,385
circ-CAMK2A	Matrix remodeling	Upregulate	miR-615-5p/ FN1	Lung adenocarcinoma	31,889,960
circ_RPPH1	Matrix remodeling	Upregulate	miR-627-5p/ miR-663a/SDC1	Glioma	35,334,040
circ_0058063	Matrix remodeling	Upregulate	miR-330-3p/ SDC4	Thyroid cancer	35,324,533
hsa_circRNA_ 102,002	Matrix remodeling	Upregulate	miR-488-3p/ HAS2	Thyroid cancer	32,862,195
circMMP1	Matrix remodeling	Upregulate	miR-1238/ MMP1/MMP2/ MMP9	Colorectal cancer	34,532,478
hsa_circRNA_ 101,996	Matrix remodeling	Upregulate	miR-143/TET2	Gastric cancer	34,659,556
ciRS-7	Matrix remodeling	Upregulate	miR-1299/MMPs	TNBC	30,072,582
circCUL2	Promote migration	Upregulate	miR-203a-3p/ MyD88 NF-kappaB/IL6	CAFs	35,189,958
circUBAP2	Pro-tumor	Upregulate	miR-4756/IFIT	CAFs	33,707,417
circZFR	Drug resistance	Upregulate	STAT3/NF-kb	CAFs	35,139,763
circFARP1	Drug resistance	Upregulate	miR-660-3p/LIF	CAFs	35,045,883

## CircRNAs and hypoxia

Due to the rapid and uncontrolled proliferation of tumors, nearly all solid tumors exhibit typical microenvironmental features such as inadequate blood supply or hypoxia. Hypoxia, characterized by reduced oxygen supply, is a hallmark of tumor microenvironment, prompting tumor cells to reprogram their metabolism through cytokine regulation in response to the hypoxic environment. Meanwhile, hypoxia stimulates angiogenesis, regulates fibrinolytic activity, and suppresses immune cell function, thereby remodeling the TME [69–71].

Enhancement of the glycolytic pathway is an important manifestation of tumor metabolic reprogramming, as evidenced by the Warburg effect which indicates increased glycolysis even under aerobic conditions, providing sufficient energy and nutrients for tumor growth and proliferation [72]. Previous studies have shown that circRNAs mainly participate in regulating the glycolytic metabolism process under hypoxic conditions, with lactate dehydrogenase A (LDHA) being their main target. For example, circMAT2B enhances glycolysis to promote HCC progression through the circMAT2B/miR-338-3p/PKM2 axis [73]. CircARHGAP29 increases LDHA stability by enhancing the interaction between LDHA and IGFBP2, leading to enhanced glycolytic metabolism in prostate cancer. Additionally, circARHGAP29 interacts with and stabilizes c-Myc expression, further increasing LDHA by promoting its transcriptional expression [74]. Similarly, in PDAC, hypoxia-induced exosomal circPDK1 which regulates the miR-628-3p/BPTF axis and degrades BIN1 to promote c-Myc activation [75].

Increased expression of HIF-1α plays a pivotal role in cellular mechanisms triggered by hypoxia. In hypoxic conditions, activated HIF-1α regulates the activity of transcription factors to downregulate E-cadherin expression, thereby promoting epithelial-mesenchymal transition (EMT) [76]. Additionally, HIF-1α disrupts the expression of enzymes involved in collagen polymerization and alignment as well as integrin activity, facilitating tumor migration. Moreover, HIF-1α mediates vascular and lymphatic vessel leakage and compression, enabling metastatic cancer cells to traverse the vessel wall [77, 78]. CircRNAs enhance the expression of HIF-1α by competitively binding to miRNAs and relieving their inhibitory effect on HIF-1α. Circ-HIPK3 upregulates the expression of HIF-1α by adsorbing miR-338-3p, and HIF-1α mediates EMT to promote the growth and metastasis of cervical cancer cells [79]. Additionally, circ-Erbin acts as a sponge for miR-125a-5p and miR-138-5p, targeting eukaryotic translation initiation factor 4E-binding protein 1 (4EBP-1), accelerating cap-independent protein translation of HIF-1α in CRC cells, and promoting angiogenesis [80]. Moreover, CircDNMT1 targets the miR576 -3p/HIF-l α axis to promote malignant behavior and metabolic reprogramming in gastric cancer [81].

CircRNAs also participate in immune regulation under hypoxic conditions, facilitating tumor cells to evade the cytotoxic effects of immune cells by inducing M2 polarization of macrophages, suppressing T cell infiltration, and enhancing the expression of tumor PD-L1, for instance, in esophageal squamous cell carcinoma, hsa-circ-0048117 is significantly upregulated and enriched in exosomes secreted by tumor cells after hypoxia preconditioning, hsa-circ-0048117 acts as a sponge for miR-140, promoting macrophage polarization towards the M2 phenotype by competing with TLR4 [82]. In hepatocellular carcinoma under hypoxic conditions, circPRDM4 acts as a scaffold to recruit HIF-1α to the CD274 promoter and consolidates their interaction, ultimately facilitating HIF- 1α-mediated transactivation of PD-L1 while inhibiting CD8 + T-cell infiltration into the tumor microenvironment and promoting immune escape [83] (Table 3).

**Table 3:** Summary of circRNAs and their functions under hypoxic conditions

circRNAs	Functions	Expression	Targets	Origin	PMID
circMAT2B	Regulate glycolysis	Upregulate	miR-338-3p/ PKM2	Hepatocellular cancer	31004447
circARHGAP29	Regulate glycolysis	Upregulate	IGF2BP2/ c-Myc/LDHA	Prostate cancer	34965937
circPDK1	Regulate glycolysis	Upregulate	miR-628-3p/ BPTF	PDAC	36068586
circ-HIPK3	Pro-tumor	Upregulate	miR-338-3p/ HIF-1α	Cervical cancer	32021434
circ-Erbin	Angiogenesis	Upregulate	miR-138-5p/ 4EBP-1/ HIF-1α	Colorectal cancer	33225938
circDNMT1	Pro-tumor	Upregulate	miR-576-3p/ HIF-1α	Gastric cancer	35712504
hsa-circ-0048117	M2 Macrophage polarization	Upregulate	miR-140/TLR4	Esophageal squamous cell carcinoma	33239890
circPRDM4	Inhibit CD8 + T cell	Upregulate	HIF-1/PD-L1	Hepatocellular carcinoma	36747292

### CircRNAs and tumor symbiotic microorganisms

Microorganisms in tumor symbiosis also exert influence on tumor growth and the remodeling of the tumor microenvironment, as they are an integral part of some tumors. A meta-analysis has revealed that approximately 15% of cancers can be directly attributed to infections caused by various etiologies such as viruses, bacteria, and parasites [84]. Furthermore, these cancers are associated with chronic inflammation, which supports the growing link between infection, inflammation, and cancer. Viral non-coding RNAs are increasingly being recognized as important regulators of infection and pathogenic mediators.

The prolonged stimulation of pathogenic bacteria results in chronic inflammation within the body, creating an immunosuppressive environment that facilitates tumor development. Tumor commensal microorganisms regulate abnormal gene expression and promote tumor progression through their own encoded circRNAs or by inducing circRNAs encoded by the tumor. Helicobacter pylori (H. pylori) is a major etiological factor in gastric cancer, and it has been shown that H. pylori infection induces circMAN1A2 expression in gastric cancer cells, promoting gastric cancer progression by acting as a sponge for miR-1236-3p and regulating MTA [85]. Additionally, H. pylori infection can increase the migration and invasion ability of gastric cancer cells by promoting the expression of circFNDC3B [86]. Hepatitis B virus (HBV) is the primary cause of hepatocellular carcinoma (HCC), and circRNAs are closely associated with HBV-induced HCC. CircBACH1 expression is elevated in both HCC tissues and HBV-transfected hepatocellular carcinoma cells, where it regulates HBV replication and hepatocellular carcinoma progression via the miR-200a-3p/MAP3K2 pathway [87]. In cervical cancer, circE7 derived from HPV16 can encode oncoprotein E7 to promote tumor growth [88]. Moreover, commensal microorganisms-encoded circRNAs also impact tumor angiogenesis. For instance, in EBV-associated gastric cancer (EBVaGC), the expression of EBV-encoded circLMP2A is positively correlated with distant metastasis and poor prognosis under hypoxic conditions due to a positive feedback loop between HIF1α and EBV-circLMP2A that promotes angiogenesis [89]. Kaposi’s sarcoma, which is commonly observed in AIDS patients, is an aggressive vascular tumor of endothelial origin caused by the oncogenic KSHV. The viral interferon regulatory factor 1 (vIRF1) encoded by KSHV induces circARFGEF1 transcription through binding to lymphoid enhancer-binding factor 1 (Lef1), thereby promoting cell motility, proliferation, and angiogenesis in vivo [90]. Merkel cell polyomavirus (MCV) expresses circRNAs, with MCV being responsible for approximately 80% of Merkel cell carcinomas (MCCs). Among these circRNAs, circMCV-T derived from MCV plays a crucial role in regulating tumor functions and viral replication [91].

Notably, the gut microbiota modulates the host’s response to cancer therapies, the composition of gut microbiota has demonstrated predictive and prognostic implications for the response to immune checkpoint blockade (ICB) therapy. The development and implementation of therapeutic strategies targeting microbial communities aim to modulate patient’s gut microbiota and its functionality, thereby optimizing clinical response to ICB treatment while minimizing treatment-related toxicity [92]. Zhu et al. demonstrated in a mouse model that SPF mice or Bifidobacterium cecum transplanted into germ-free mice significantly inhibited lung metastasis. Additionally, significant differences were observed in circRNA and miRNA expression between germ-free and SPF mice transplanted with Bifidobacterium cecum. It was found that the mmu_circ_000073/mmu-miR-466i-3p/SOX9 axis could promote EMT (epithelial-mesenchymal transition) and tumor metastasis [93]. From this, it can be seen that abnormal expression of circRNAs may be one of the reasons for tumor progression caused by dysregulated gut microbiota, and regulating the expression of circRNAs in the gut microbiota could potentially become a means of cancer treatment (Table 4).

**Table 4:** Summary of circRNAs and their functions in tumor symbiotic microorganisms

circRNAs	Functions	Expression	Targets	Microorganisms	PMID
circMAN1A2	Pro-tumor	Upregulate	miR-1236-3p/ MTA2	H. pylori	35,484,118
circFNDC3B	Pro-tumor	Upregulate	miR-942/CD44, miR-510/CDH1	H. pylori	35,418,175
circBACH1	Pro-tumor	Upregulate	miR-200a-3p/ MAP3K2	HBV	35,352,818
circE7	Pro-tumor	Upregulate	/	HPV16	31,127,091
EBV-circLMP2A	Angiogenesis	Upregulate	KHSRP/VHL/HIF-1a/VEGFA	EBV	34,863,886
circARFGEF1	Pro-tumor/ Angiogenesis	Upregulate	miR-125a-3p/ GLRX3	KSHV	33,539,420
circMCV-T	Pro-tumor	Upregulate	/	MCV	33,323,517
mmu_circ_ 000073	Pro-tumor	downregulate	mmu-miR-466i-3p/SOX9	Gut microbiota	32,686,598

### CircRNAs and immunosuppression

As the most abundant cellular component of the TME, immune cells play pro- or anti-tumorigenic roles in the tumor microenvironment. Immune cells undergo three stages of immune editing within the TME: elimination, homeostasis, and escape, in which tumor cells evade the host’s immune response by shedding surface antigens or down-regulating the expression of key molecules required for interaction with immune cells [2, 94]. In addition, tumor cells actively recruit lymphocytes, myeloid suppressor cells, macrophages, etc. to the tumor site through the production of chemokines. Tumor-associated immune cells produce cytokines and growth factors that are essential for tumor growth but do not exert anti-tumor functions. Immunotherapy targeting immune checkpoints such as PD-1/PD-L1 as well as CTLA- 4 restores the activity of depleted CD8 + T cells to kill tumor cells, resulting in good overall survival in mutation-negative patients [95, 96]. However, not all patients derive benefit from immunotherapy and relapse is an inevitable occurrence in those receiving this treatment modality. therefore, further elucidation of the regulatory mechanisms governing immune cells within tumors remains imperative. circRNAs exert a pivotal role in regulating the functions of tumor-associated macrophages (TAMs), regulatory T (Treg) cells, CD8 + T cells, and NK cells (Table 5).

### CircRNAs and macrophages

Macrophages are a heterogeneous group of immune cells that differentiate into distinct phenotypes and exhibit specific biological functions in response to various stimuli, including cytokines, growth factors, inflammation, infection, injury, hypoxia, and other conditions [97]. These cells can be classified as either classically activated M1 or alternatively activated M2 macrophages. however, the phenotypes of these subtypes can be interchanged depending on the stimulatory factors present [98]. While M1-type macrophages display pro-inflammatory effects, M2-type macrophages possess anti-inflammatory properties and promote wound healing as well as vascular-lymphangiogenic functions. Additionally, tumor-associated macrophages (TAMs), which share similarities with M2-like macrophages in terms of their phenotype and function profile, play a crucial role in promoting tumorigenesis [99].

CircRNAs exert a positive regulatory role in modulating macrophage function, and circRNAs derived from tumor cells can induce M2 polarization of macrophages [100]. Extensive studies have demonstrated that the polarization of macrophages is governed by signaling pathways such as JAK1/STAT3 and PI3K-AKT, wherein circRNAs actively participate [101–104]. For instance, in ovarian cancer, exosome-derived circATP2B4 can be delivered to infiltrating macrophages and induce M2-type polarization through modulation of the miR-532-3p/SREBF1/PI3Kα/AKT axis, leading to immunosuppression and ovarian cancer metastasis [105]. Exosomes containing circFARSA mediate M2-type polarization of macrophages through the PTEN/PI3K/AKT pathway and promote EMT and metastasis in non-small cell lung cancer [106]. In breast cancer, endoplasmic reticulum stress promotes the secretion of tumor-derived exosomes and enhances the entry of circ_0001142 into macrophages, which interferes with macrophage autophagy and polarization processes by miR-361-3p/PIK3CB axis [107]. The exosomes circSAFB2 promotes kidney cancer metastasis by mediating M2-type macrophage polarization through the miR-620/JAK1/STAT3 axis [108]. Hsa-circ-0048117 is significantly upregulated and enriched in exosomes secreted by esophageal squamous cell carcinoma after hypoxic preconditioning, and it competes with TLR4 to adsorb mir-140, promoting macrophage polarization towards the M2 phenotype [82]. It is worth noting that the phenotype of macrophages can be converted under different cytokine stimuli, inducing the transformation of tumor-associated macrophages into tumor-killing cells, which has become an important approach in immunotherapy [109], and circRNAs may serve as a potential target for this purpose.

M2 macrophages that differentiate in response to cytokines within the tumor microenvironment lose their anti-tumor efficacy and secrete immunosuppressive factors such as IL-10, TGF-β, and indoleamine 2,3-dioxygenase (IDO), thereby facilitating immune evasion [110]. Furthermore, exosomes circRNAs derived from macrophages also actively participate in various physiological processes that promote tumor growth. For instance, RBPJ + macrophage-secreted exosomes containing hsa_circ_0004658 impede the progression of hepatocellular carcinoma through the miR-499b-5p/JAM3 pathway [111]. In cutaneous squamous cell carcinoma, M2-type macrophages upregulate circ_TNFRSF21 to promote angiogenesis by competitively adsorbing miR-3619-5p and increasing ROCK expression [112]. Furthermore, TAMs-derived exosomes transfer hsa_circ_0001610 to endometrial cancer cells and enhance cyclin B1 expression by adsorbing miR-139-5p, thereby reducing the radiosensitivity of endometrial cancer cells [113]. Additionally, the exosomes circZNF451 inhibit anti-PD1 therapy in lung adenocarcinoma by polarizing macrophages in complex with TRIM56 and FXR1 [114].

### CircRNAs and T lymphocytes

Tumor-infiltrating lymphocytes (TIL) are an integral part of the host’s inflammatory response to tumors. However, mounting evidence suggests that despite their activating phenotype, TIL exhibit functional impairment due to the absence of Th1 cytokines (such as IL-2, IFN-γ, and IL-12) and the prevalence of Treg cells cytokines (such as IDO or TGF-β) at tumor sites. This conversion leads to a Th2 or Treg functional phenotype in tumor-specific T-cells. Furthermore, Treg cells impede effector T cell infiltration and CD8 + T cell cytotoxicity while promoting cancer cell survival [115]. CircRNAs also play a regulatory role in tumor-infiltrating lymphocytes (TILs), as evidenced by the ability of HCC cell-derived exosomes circGSE1 to induce Treg cell expansion through modulation of the miR-324-5p/TGFBR1/Smad3 axis [116]. Hsa_circ_0136666 to regulate Treg cells activity via targeting the miR-497/PD-L1 axis in colorectal cancer [117], and cancer cell-derived exosomes circUSP7 to inhibit CD8 + T cell secretion of IFN-γ, TNF-α, granzyme-b, and perforin by adsorbing miR-934 to up-regulate protein tyrosine phosphatase 2 (SH2)-containing Src homology region 2 (SH2) expression in non-small cell lung cancer [118]. Additionally, bladder cancer cells release exosome-derived circTRPS1 that regulates intracellular reactive oxygen species homeostasis and CD8 + T cell depletion via the circTRPS1/miR141-3p/GLS1 axis [119]. It has been demonstrated that methylation modifications play a crucial role in regulating the expression, function, and stability of circRNAs [120]. Additionally, methylated circRNAs have been shown to be involved in tumor immunity regulation. In NSCLC, N(6)-methyladenosine-modified circIGF2BP3 inhibits CD8 + T-cell responses by promoting deubiquitination of PD-L1, thereby facilitating tumor immune escape [121]. These findings provide novel insights into potential immunotherapeutic targets.

### CircRNAs and NK cells

NK cells are innate immune cells that protect cells from immune attack by recognizing MHC class I molecules on the surface of normal cells. Tumor cells are recognized by NK cells due to the loss of MHC class I molecules, and NK cells play a role in tumor immunosurveillance by directly killing or releasing cytokines to clear newly arising tumors or metastases without prior sensitization. Developing tumors utilize multiple mechanisms to evade NK cell-mediated immune surveillance, resulting in limited access of NK cells to the tumor bed, altered NK cell phenotype and function, and loss of immunogenicity, which impedes recognition of tumor cells by NK cell receptors [122, 123]. The expression of immune-related molecules, such as PD-L1 and ICAM-1, is regulated by circRNAs through ceRNA networks, leading to the inhibition of NK cell activity and acceleration of their exhaustion. Consequently, this facilitates tumor immune evasion. For example, circFOXO3, hsa_circ_0048674, hsa_circ_0007456, and circRHOT1 affect the activity of NK cells by adsorbing miRNAs, causing NK cell senescence and promoting tumor growth, respectively [124–127].

**Table 5:** Summary of circRNAs and their functions in immunosuppression

circRNAs	Functions	Expression		Targets	Origin	PMID
circATP2B4	M2 Macrophage polarization	Upregulate	miR-532-3p/ SREBF1		Ovarian cancer	36,512,324
circFARSA	M2 Macrophage polarization	Upregulate	PTEN/PI3K/AKT		Lung cancer	34,119,765
circ_0001142	M2 Macrophage polarization	Upregulate	miR-361-3p/ PIK3CB		Breast cancer	35,091,089
circSAFB2	M2 Macrophage polarization	Upregulate	miR-620/ JAK1/STAT3		Kidney cancer	35,646,637
hsa-circ-0048117	M2 Macrophage polarization	Upregulate	miR-140/TLR4		Esophageal squamous cell carcinoma	33,239,890
hsa_circ_0004658	Inhibit tumor	Upregulate	miR-499b-5p/ JAM3		Macrophage	35,013,102
hsa_circ_0017252	M2 Macrophage polarization	Upregulate	miR-17-5p		Gastric cancer	35,796,939
hsa_circ_0001610	Drug resistance	Upregulate	miR-139-5p/ cyclin B1		Macrophage	34,462,422
circRNA TCFL5	M2 Macrophage polarization	Upregulate	miR-543/FMNL2		Esophageal cancer	35,646,112
circPCLE1	M2 Macrophage polarization	Upregulate	miR-485-5p/ ACTG1		Colorectal cancer	35,349,390
hsa_circ_0094606	M2 Macrophage polarization	Upregulate	PRMT1/ILF3/ IL-8		Prostate cancer	36,394,342
hsa_circ_0003410	Recruit M2 Macrophage	Upregulate	miR-139-3p/ CCL5		Hepatocellular cancer	34,890,089
circSMARCC1	M2 Macrophage polarization	Upregulate	miR-1322/ CCL20/CCR6		Prostate cancer	36,045,408
circ_0018909	M2 Macrophage polarization	Upregulate	miR-545-3p/ FASN		Pancreatic cancer	36,541,402
circZNF451	M2 Macrophage polarization	Upregulate	FXR1/ELF4/IRF4		Lung adenocarcinoma	36,209,117
hsa_circ_0074854	M2 Macrophage polarization	Downregulate	HuR		Hepatocellular carcinoma	33,880,025
circHIPK3	M2 Macrophage polarization	Upregulate	PTK2		Lung cancer	34,326,381
circNEIL3	M2 Macrophage polarization	Upregulate	IGF2BP3		Glioma	35,031,058
circITGB6	M2 Macrophage polarization	Upregulate	/		Ovarian cancer	35,277,458
circGSE1	Regulate Tregs	Upregulate	miR-324-5p/ TGFBR1/Smad3		Hepatocellular carcinoma	35,396,771
hsa_circ_0136666	Regulate Tregs	Upregulate	miR-497/PD-L1		Colorectal cancer	34,320,370
circUSP7	Inhibit CD8 + T cell	Upregulate	miR-934/SHP2		Lung cancer	34,753,486
circTRPS1	Inhibit CD8 + T cell	Upregulate	miR141-3p/GLS1		Bladder cancer	35,038,580
circIGF2BP3	Inhibit CD8 + T cell	Upregulate	miR-328-3p、 miR-3173-5p/ PKP3		Lung cancer	34,416,901
circFOXO3	Inhibit NK cell	Upregulate	miR-29a-3p、miR-122-5p/ KLF16		Renal cancer	36,072,902
hsa_circ_0048674	Inhibit NK cell	Upregulate	miR-223-3p/ PDL1		Hepatocellular carcinoma	35,187,630
hsa_circ_0007456	Inhibit NK cell	Upregulate	miR-6852-3p/ ICAM-1		Hepatocellular carcinoma	33,462,208
circRHOT1	Inhibit NK cell	Upregulate	miR-3666/ SMAD5		Bladder cancer	34,926,705

### Future remarks

With a comprehensive understanding of the structure and function of circRNAs, they have emerged as a crucial player in tumorigenesis, development, and regulation of the tumor microenvironment. As such, circRNAs represent an exciting area of research in cancer biology with potential implications for therapeutic interventions. By exploring the interactions between circRNAs and key components of TME, we propose that circRNAs hold promise as both diagnostic biomarkers and therapeutic targets.
